# Effect of a Community-Based Program on Preschoolers’ Physical Activity and Nutrition in Chile

**DOI:** 10.3390/jfmk10010093

**Published:** 2025-03-12

**Authors:** Gabriela Salazar, Fabian Vasquez, Margarita Andrade, Maria del Pilar Rodriguez, Rocio Berlanga, Juanita Rojas, Antonio Giadalah, Alvaro Muñoz

**Affiliations:** 1 Institute of Nutrition and Food Technology (INTA), University of Chile, Santiago 7800284, Chile; 2 School of Nutrition and Dietetic, Finis Terrae University, Santiago 7501014, Chile; 3 School of Nutrition and Dietetic, University of Chile, Santiago 7800284, Chile; 4 National Daycare Centers (JUNJI), Santiago 7800284, Chile; 5 National Institute of Sports, Santiago 7501014, Chile

**Keywords:** childhood obesity, physical activity, dietary habits, community intervention, daycare centers, Chile

## Abstract

**Introduction:** Childhood obesity has reached critical levels in Chile, particularly among preschoolers from low-income families who face barriers to nutritious food and physical activity. Early interventions are essential to mitigate long-term health risks. This study evaluates the Chile Active Intervention, a community-based program promoting physical activity and healthy eating among preschoolers attending public daycare centers in Antofagasta, Santiago, and Temuco. **Objective:** To assess the effectiveness of a structured intervention in improving physical activity levels, dietary habits, and obesity-related risk factors in children aged 3 to 5 years old. **Methods:** A quasi-experimental design was implemented with intervention and control groups, including 1204 children from public daycare centers. The intervention-comprised educator training on healthy eating structured physical activity sessions tailored for young children and family engagement through “Healthy Days” events. Pre- and post-intervention assessments measured anthropometric variables, body composition, physical activity, and dietary intake. **Results:** The intervention led to positive changes in weight-for-height Z-scores, body fat percentage, and skinfold thickness, particularly among high-risk children. Physical activity assessments showed reduced sedentary time and increased active play. Dietary improvements included higher fruit and vegetable consumption and reduced ultra-processed food intake. **Conclusions:** This study demonstrates that early, community-based interventions can effectively improve health behaviors in preschoolers. The program’s scalability across Chile is promising, with parental involvement and institutional support being key to sustaining impact. Long-term evaluations are recommended to assess its lasting effects on childhood health outcomes.

## 1. Introduction

Childhood obesity has emerged as a critical global health concern, with prevalence reaching alarming levels in both developed and developing nations. According to the World Health Organization (WHO), more than 39 million children under the age of five were overweight or obese in 2020, reflecting a public health crisis with substantial long-term consequences [1]. This growing epidemic is driven by a combination of behavioral, socioeconomic, and environmental factors, as well as the nutritional transition marked by a shift toward diets rich in ultra-processed foods, saturated fats, sugars, and sodium [2,3]. These changes, exacerbated by globalization and urbanization, are particularly acute in low- and middle-income countries (LMICs), where traditional dietary habits are rapidly being replaced [4].

The Latin American context and Chile’s growing obesity epidemic

In Latin America, a region that grapples with the double burden of malnutrition—where undernutrition coexists with obesity—the rise in childhood obesity is particularly alarming [5]. Chile stands out as a clear example, exhibiting some of the highest childhood obesity rates in the region. The National Health Survey (2017) reported that one-third of children under five were overweight or obese, a trend that has seen sustained increases over the past two decades [3]. Similar patterns have been observed in other Latin American nations, including Mexico and Brazil, where urbanization, increased exposure to processed foods, and reduced physical activity levels have converged to exacerbate the issue [6,7,8].

Determinants of Childhood Obesity

Childhood obesity is influenced by a multifactorial interplay of genetic, environmental, and behavioral factors. Environmental contributors, such as limited access to nutritious foods and safe spaces for physical activity, are particularly prevalent in low-income urban settings [9]. Research has highlighted that food insecurity and food deserts—areas where access to fresh products is limited—significantly increase the consumption of energy-dense, nutrient-poor foods [10,11]. Social determinants such as parental education levels, family income, and cultural attitudes toward food and physical activity further amplify these challenges [12].

In the Chilean context, the implementation of the Food Labeling and Advertising Law (2016) has demonstrated measurable success in reducing the consumption of high-sugar and high-fat products [13]. However, despite these advances, systemic barriers remain, particularly in communities with limited resources to support sustained lifestyle changes. This underscores the need for integrated community-based interventions that address the broader social and environmental determinants of obesity.

Community- and school-based interventions: a global perspective

Community-based and school-based interventions have proven to be highly effective in combating childhood obesity through a combination of nutrition education, structured physical activity, and parental involvement. Globally, multicomponent programs have yielded promising results:

United States: The “Hip-Hop to Health” program demonstrated that culturally adapted interventions combining education and physical activity could significantly reduce BMI and promote healthier lifestyles in preschool-aged children from minority backgrounds [14,15].

Australia and Europe: Programs implemented in early childhood education settings have shown that structured interventions focusing on physical activity, healthy eating, and parental participation lead to reductions in BMI Z-scores and improvements in body composition [16,17]. Notably, the InFANT program in Australia emphasized parental education as a key strategy for creating sustainable dietary and physical activity changes [18].

Latin America: Countries like Brazil and Mexico have implemented culturally relevant interventions that address both dietary and activity behaviors, with programs like Agita São Paulo emphasizing the importance of physical activity in reducing sedentary lifestyles [19,20].

A systematic review conducted by Hesketh & Campbell (2010) confirmed that interventions within childcare settings that integrate both structured physical activity and healthy food environments are among the most effective strategies for reducing childhood obesity globally [21].

The role of nutrition education in obesity prevention

The role of nutrition education is central to the success of community-based interventions. Behavioral strategies that target both children and their caregivers have demonstrated long-term benefits in improving dietary habits. Recent studies emphasize that parent-focused interventions are particularly impactful, as parents serve as primary role models and decision-makers for their children’s diets [22,23]. Programs that provide hands-on education about meal preparation, portion sizes, and the benefits of fresh products have been linked to increased fruit and vegetable consumption and reduced intake of sugar-sweetened beverages [24,25].

Furthermore, schools and daycare centers play a pivotal role as environments where healthy behaviors can be reinforced. Policies restricting access to unhealthy foods within school settings and mandating the provision of nutrient-dense meals have been shown to reduce obesity prevalence among school-aged children [26]. Programs such as Water First in Canada and the Healthy Start initiative in the UK exemplify the importance of creating supportive food environments that align with educational efforts [27,28].

The critical role of physical activity

Physical activity remains a cornerstone of childhood obesity prevention. Structured programs that promote moderate-to-vigorous physical activity (MVPA) have demonstrated clear benefits not only in weight management but also in improving motor development, cognitive function, and emotional well-being [29]. The WHO recommends at least 180 min of daily physical activity for children under five, distributed across multiple intensities [30]. Studies have shown that integrating active play and structured movement programs within school curricula leads to significant reductions in sedentary behavior and BMI levels [31].

However, research highlights barriers to the implementation of these recommendations, particularly in low-resource settings where access to safe play spaces and structured exercise programs is limited [32]. Addressing these barriers requires a coordinated effort involving policymakers, educators, and community leaders to invest in infrastructure and promote equity in physical activity opportunities.

The need for community-based interventions in Chile

While Chile has made significant strides through policy-driven approaches, such as food labeling laws, there remains a critical need for community-based interventions that address both dietary and physical activity behaviors during early childhood. Schools and daycare centers provide a unique opportunity to target these behaviors at a formative stage, as children spend substantial time in these environments [33,34].

The present study evaluates a multicomponent intervention implemented in three Chilean cities—Antofagasta, Santiago, and Temuco—representing diverse socioeconomic and geographic contexts. The program combines nutrition education, structured physical activity, and community engagement, aligning with global best practices and addressing the multifactorial determinants of childhood obesity. This approach reflects the growing consensus that holistic, culturally adapted interventions are essential to effectively combat childhood obesity [35,36].

By contributing to the global evidence base, this study provides a scalable and replicable model that can be applied in other Latin American countries and similar resource-constrained settings worldwide.

## 2. Materials and Methods

### 2.1. Study Design

This study employed a quasi-experimental design with intervention and control groups to evaluate the effectiveness of a multicomponent intervention targeting childhood obesity. The intervention was conducted in three Chilean cities: Antofagasta (northern desert region), Santiago (central Metropolitan region), and Temuco (southern forest region). These cities were selected to represent diverse geographic, socioeconomic, and cultural contexts, thereby enhancing the generalizability of findings. The study was conducted over 4 months, with evaluations conducted at baseline and post-intervention.

Daycare centers were randomly assigned to either the intervention or the control group using a stratified randomization approach to ensure balanced allocation based on geographic region, socioeconomic characteristics, and enrollment size. A computer-generated randomization sequence was used, and an independent researcher not involved in data collection conducted the assignment. This process minimized potential selection bias and ensured comparability between groups.

The control group continued with their standard practices, while the intervention group received a structured program focusing on nutritional education, physical activity, and community participation.

### 2.2. Subjects

The number of children who participated was n = 1204, distributed in n = 265 in Phase 1 and n = 939 in Phase 2. The overall dropout rate was 7.8%, with reasons for withdrawal including relocation, change in daycare enrollment, withdrawal of consent, and insufficient attendance (<80%). Missing data were handled using multiple imputation techniques, ensuring the robustness of the findings. The distribution of participants across the two phases was determined by several factors, including logistical constraints, program scalability, and lessons learned from the initial phase. Phase 1 served as a pilot study, focusing on a smaller subset of children in Santiago to test the feasibility, acceptability, and effectiveness of the intervention before expanding to a larger scale. The findings from Phase 1 were used to refine intervention strategies, improve educator training, and optimize engagement methods for children and parents.

Phase 2 represented a significant expansion, including a much larger cohort across three geographically diverse regions: Antofagasta (North), Santiago (Central), and Temuco (South). This phase aimed to assess the program’s scalability, regional adaptability, and overall effectiveness across different socioeconomic and environmental contexts. The increased sample size in Phase 2 allowed for greater statistical power and more representative insights into the intervention’s impact across diverse populations. Additionally, logistical and administrative barriers encountered during Phase 1 were addressed in Phase 2, facilitating smoother participant recruitment. More daycare centers participated in the second phase due to increased institutional support and greater awareness of the intervention’s benefits among educators and parents.

The inclusion and exclusion criteria were: children were eligible for the study if they were between 3 and 5 years old and enrolled in public daycare centers participating in government social programs. Regular attendance at daycare was required, defined as at least 80% attendance in the 2 months prior to the intervention. Additionally, parental or guardian informed consent was mandatory for participation in physical activity sessions, dietary assessments, and anthropometric evaluations. Only children with no known medical conditions restricting physical activity were included.

Exclusion criteria were established to ensure the reliability of the study. Children with diagnosed chronic diseases, such as congenital heart disease, endocrine disorders (e.g., diabetes), or other metabolic conditions that could interfere with weight gain/loss patterns, were excluded. Participants receiving special dietary interventions for medical reasons were also ineligible. Furthermore, children whose parents did not provide written informed consent or those who withdrew from the study before baseline assessments were not included. Lastly, participants with incomplete baseline data, making it impossible to track changes throughout the intervention, were also excluded from the final analysis.

This study was conducted in accordance with the ethical principles outlined in the Declaration of Helsinki. The Ethics Committee of Universidad de Chile granted ethical approval. Written informed consent was obtained from the parents or legal guardians of all participating children before their enrollment in the study.

### 2.3. Intervention

The Social Cognitive Theory (SCT) and the Ecological Model of Health Behavior served as the foundational frameworks for this intervention. SCT, developed by Bandura, posits that behavior change is influenced by the dynamic interaction between personal, behavioral, and environmental factors, emphasizing self-efficacy, observational learning, and reinforcement as key drivers of sustained health behaviors [37]. In this intervention, SCT principles were applied through educator training, modeling of healthy behaviors, and structured reinforcement to encourage preschoolers’ physical activity and healthy eating habits. The Ecological Model, on the other hand, recognizes the role of multiple layers of influence, including individual, interpersonal, institutional, community, and policy-level factors on health behaviors [38]. This model justified the inclusion of family engagement (“Healthy Days”), modifications to the daycare environment, and community-wide efforts to promote physical activity and healthy eating in preschool settings.

The intervention consisted of three main components:

▪ Nutritional education: Kindergarten educators were trained through participatory workshops on healthy eating habits and the importance of fruit and vegetable consumption. Educational materials were also designed to support daily activities in the daycare centers [16]. The design of the Didactic Guides for Physical Activity and Nutrition was based on the curricula of preschool education. Didactic Guides contained 45–50 games to be played daily with the children, aiming to increase physical activity as well as incorporate healthy food habits knowledge. The educational training was directed to educators and technicians; motivational workshops were conducted with the parents. Nutritionists and physical activity teachers trained all participant educators for 2 months, during 3-day workshops, to favour the participation of all educators. They also visited each other three times a week at each intervention centre. An active and motivational strategy was designed for parents, Healthy Days, once a month, consisting of participative games, healthy snacks, and leaflets with information on growth, nutritional factors, and physical activity needs.
▪ Promotion of physical activity: Age-appropriate, playful physical activities were developed to improve children’s levels of moderate-to-vigorous physical activity [24]. Daily exercise sessions and outdoor games were included. The specific PA strategies included:
  ▪ Daily active play sessions lasting at least 60 min per day, broken into three to four 15–20-min blocks throughout the school day.
  ▪ Integration of movement into learning activities, such as using dance, stretching, and action-based storytelling to reinforce educational content.
  ▪ Reduction of prolonged sitting, ensuring that sedentary periods did not exceed 30 min at a time.
  ▪ Outdoor free play sessions encouraging children to explore their environment and engage in self-directed physical activity.
  ▪ Encouragement of home-based PA, with educators providing parents with recommendations for simple activities that could be done with minimal equipment.
▪ Community participation: Community activities, such as “healthy days,” were organized to engage parents and promote the adoption of healthy habits at home [39].

The implementation of this methodology in Phase 1 included:

▪ Educative diagnostic
▪ The design and validation of educative methodology
▪ The design and validation of didactic material for educators in both nutrition and physical activity aspects was designed to facilitate their activities with children.

In the second phase, the validated methodology was applied to preschool education in daycare centers and schools in three different cities in Chile. The selection of cities was based on cities with a higher prevalence of obese children.

### 2.4. Evaluation

Pre- and post-intervention evaluations were conducted, including the following measurements.

Anthropometric variables

Nutritionists trained in standardized protocols for assessing body dimensions conducted anthropometric measurements. Weight was measured to the nearest 10 g using a SECA digital scale (Model 770, SECA^®^, Hamburg, Germany), ensuring precision. Height was measured to the nearest 0.1 cm using a portable Holtain stadiometer (Model 603, Holtain Ltd., Crosswell, UK). The measurement procedures followed the established methodologies of the third U.S. National Health and Nutrition Examination Survey (NHANES III) [40].

Skinfold thickness at four anatomical sites (biceps, triceps, subscapular, and suprailiac) was measured using a Lange caliper with millimeter precision (1 mm). Triplicate measurements were performed for accuracy, following the protocols outlined by Lohman et al. (1984) [40]. The intra-observer technical error of measurement and mean observer bias fell within the acceptable limits established by the World Health Organization [41].

Body composition

Total body water (TBW) was assessed using the deuterium dilution technique, a recognized gold standard for measuring body composition. Participants received a dose of deuterium-labeled water equivalent to 0.4 g per kilogram of body weight (98.9% atom excess; Europa Scientific, Cambridge, UK). A baseline saliva sample (3 mL) was collected prior to dosing to establish pre-dose deuterium abundance. Post-dose saliva samples were collected 3–4 h after ingestion using sterile cotton swabs designed for absorbent saliva collection. All samples were stored at −20 °C for later analysis. TBW was quantified using the equilibration method on a HYDRA isotope ratio mass spectrometer (HYDRA IR-MS; Europa Scientific). Each sample was analyzed in triplicate to ensure accuracy and minimize variability. The method demonstrated a coefficient of variation of 1.2% and precision levels of 2–3%, confirming its reliability for body composition analysis in research settings [42].

Physical activity

A triaxial movement sensor (TRITRAC-R3D RESEARCH ERGOMETER, Professional Products, Division of Reining International, Madison, WI, USA) was employed to capture detailed information on three-dimensional physical activity, including lateral, vertical, and horizontal movements. These data were collected every minute and stored as a Vector of Magnitude (VM), calculated as the square root of the sum of squared movement vectors along the X, Y, and Z axes. The collected data were subsequently downloaded to a computer for analysis, providing a comprehensive overview of physical activity patterns during the monitoring period [43].

The Tritrac sensor is particularly effective in estimating energy expenditure from physical activity in adults and has established cut-off points for categorizing moderate and vigorous activities in older children and adults. However, preschool-aged children differ from older children in several ways, including lower body weight, height, and ongoing motor skill development, necessitating distinct activity thresholds. Activity categories for preschool children were developed based on direct observation of 20 children with normal nutritional status. Interestingly, the moderate and vigorous activity thresholds identified in this group were similar to the 3 METs benchmark (~1000 counts) previously reported in older populations [43].

Children wore the Tritrac monitors continuously over 3 days, including 2 weekdays and one weekend day. The monitor was attached immediately after waking and removed at bedtime, with parents or caregivers asked to document these times and any interruptions (e.g., for showers or swimming) using a questionnaire designed for the study. To prevent tampering and ensure accurate positioning, the sensor was secured in a pocket stitched onto a chest harness. This placement minimized shifting and captured accurate activity data without restricting the child’s natural movement.

While the hip is a conventional placement for accelerometers, it proved unsuitable for this age group due to frequent manipulation or discomfort, particularly among three-year-olds. The chest placement, approximately 8–10 cm from the hip in preschool-aged children, was validated through an unpublished study comparing activity data from the Tritrac placed on the chest with heart rate monitoring and treadmill tests at variable intensities. This study confirmed that chest placement yielded reliable activity measurements in children aged 3–5 years old.

### 2.5. Statistical Analysis

Continuous variables are summarized as means ± standard deviations, while categorical variables are presented as frequencies and percentages. Data were analyzed using independent *t*-tests and paired *t*-tests to compare pre- and post-intervention means. *p*-values below 0.05 were considered statistically significant. To address missing data, mean imputation was applied for instances where the missing values constituted less than 5%, ensuring the integrity of the analyses without introducing substantial bias. For cases where missing data exceeded 5%, those entries were excluded from the respective analysis to prevent distortions. Statistical analyses were conducted using STATA software, version 18.0, with a significance threshold set at *p* < 0.05 (StataCorp, 2023, Stata Statistical Software: Release 18, StataCorp LLC, College Station, TX, USA).

## 3. Results

The results of this two-phase intervention study demonstrate the impact of community-based programs on improving physical activity and dietary habits among preschool children attending public daycare centers in various regions of Chile. The initial anthropometric assessment of the participants revealed similar baseline characteristics between the intervention and control groups, ensuring comparability at the start of the study. At the beginning of the study, both the intervention and control groups exhibited similar anthropometric characteristics, with no statistically significant differences observed between groups at baseline (*p* > 0.05 for all variables). The study included 1204 preschool children aged 3 to 5 years old (mean age 4.3 ± 0.7 years old), with a nearly equal distribution of boys (51.3%) and girls (48.7%). The average age of the children was 4.4 ± 0.4 years old in the intervention group and 4.5 ± 0.4 years old in the control group (*p* = 0.08), indicating a comparable developmental stage. Regarding weight and height, children in the intervention group had an average weight of 18.6 ± 2.9 kg, while those in the control group weighed 19.3 ± 3.1 kg (*p* = 0.06). Their height was 105.4 ± 4.6 cm and 106.6 ± 4.5 cm, respectively (*p* = 0.07). In terms of Z-scores for anthropometric indicators, the weight-for-age Z-score was slightly higher in the intervention group (0.8 ± 1.2) compared to the control (0.7 ± 1.2, *p* = 0.09), whereas the height-for-age Z-score was marginally lower in the intervention group (−0.1 ± 1.0) than in the control (0.1 ± 1.0, *p* = 0.05). The weight-for-height Z-score, a key indicator of weight status in early childhood, was 0.8 ± 1.2 in the intervention group and 0.9 ± 1.2 in the control group (*p* = 0.04). These results indicate that the two groups started from a relatively homogeneous anthropometric profile, which strengthens the reliability of subsequent comparisons assessing the impact of the intervention (Table 1). At baseline, 29.0% of the children were classified as obese, according to WHO weight-for-height Z-score standards. The sample was distributed into two phases, with 265 children participating in Phase 1 as part of a pilot implementation in Santiago, while 939 children participated in Phase 2, which expanded the intervention to Antofagasta, Santiago, and Temuco. Table 2 presents the initial anthropometric data of children categorized as obese and normoweight in the intervention and control groups across three boroughs in Santiago, Chile. Among obese children, there were 35 participants in the intervention group and 42 in the control group. No statistically significant differences were observed in age (*p* = 0.85), weight (*p* = 0.09), height (*p* = 0.07), or height-for-age Z-score (*p* = 0.92). However, a trend toward lower weight-for-height Z-scores was seen in the intervention group (2.7 ± 0.8) compared to controls (3.0 ± 0.9), though this difference was not statistically significant (*p* = 0.08). The weight-for-age Z-score was also slightly lower in the intervention group (2.2 ± 1.0 vs. 2.4 ± 0.9, *p* = 0.11), indicating comparable baseline characteristics between groups. Among normoweight children, 85 were in the intervention group, while 103 were in the control group. No significant differences were observed in age (*p* = 0.06), weight (*p* = 0.07), height (*p* = 0.09), or height-for-age Z-score (*p* = 0.33). However, a statistically significant difference was found in the weight-for-height Z-score, with children in the intervention group having a lower Z-score (0.1 ± 0.5) compared to controls (0.3 ± 0.5, *p* = 0.04). This difference suggests that at baseline, normoweight children in the control group exhibited a slightly higher tendency toward weight gain relative to height.

Overall, Table 2 provides a baseline comparison, showing similar initial anthropometric characteristics between intervention and control groups before the implementation of the intervention.

In Phase 2, the study expanded to include larger groups across three diverse regions of Chile: Antofagasta in the North, Santiago in the Metropolitan region, and Temuco in the South. Table 3 presents anthropometric data from Phase II across intervention and control groups. Among obese children, there were significant differences in weight-for-height Z-score (*p* = 0.02) and height-for-age Z-score (*p* = 0.04), with the intervention group showing improved outcomes. In the normoweight group, a statistically significant difference was also observed in height-for-age Z-score (*p* = 0.03), indicating the intervention’s impact on maintaining adequate growth patterns.

Further analysis of body composition during Phase 2 highlighted reductions in both body fat percentage and skinfold thickness among children in the intervention group. The total group in the intervention cohort showed a slight decrease in body fat from 25.0% to 24.7% (*p* = 0.04), while controls experienced an increase from 24.8% to 25.1% (*p* = 0.05). Obese children in the intervention maintained stable body fat percentages (31.6%), contrasting with a rise in the control group from 30.7% to 31.4% (*p* = 0.03). Additionally, skinfold measurements revealed a reduction in the intervention group, with a drop from 60.3 mm to 57.9 mm (*p* = 0.02), while an increase was observed in the control group (*p* = 0.05). These findings suggest that the intervention was effective in mitigating adiposity, especially among children who had higher levels of excess body fat at baseline Table 4.

**Figure 1:**
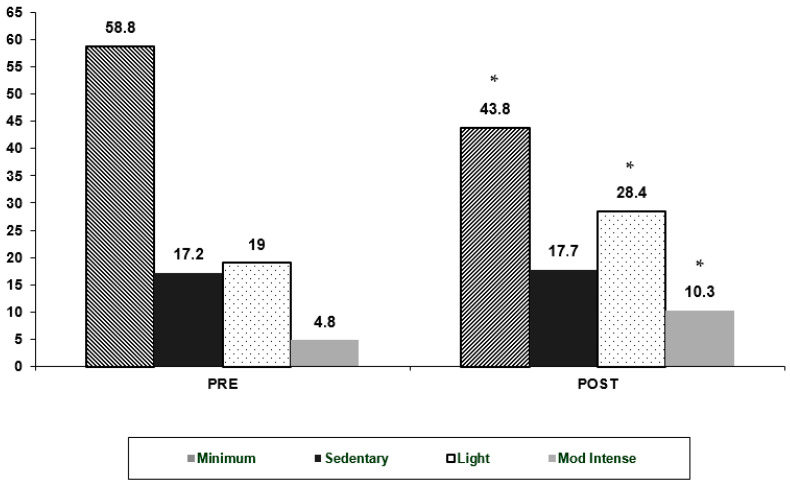
Physical activity pattern in Intervention participants, pre- and post-intervention in minimum, sedentary, light, and mod-intense activities. * Statistically significant differences at *p* ≤ 0.05.

The intervention resulted in significant changes in the physical activity levels of participating children. As illustrated in Figure 1, prior to the intervention, the majority of children engaged in minimal activity, with a high proportion classified as sedentary. Light and moderate-intensity physical activities were less frequent, indicating a predominantly inactive lifestyle among the participants. Following the intervention, a notable shift in activity patterns was observed. The proportion of children classified under minimum activity significantly decreased, while those engaging in light and moderate-intensity physical activities increased. Specifically, minimum time was significantly reduced (*p* = 0.03), and there was a statistically significant increase in light physical activity (*p* = 0.02). Additionally, moderate-intensity activity showed a significant improvement (*p* = 0.01) (Figure 1).

While specific dietary data were not included in the tables, qualitative findings indicate that dietary improvements were notable within the intervention group. Children and families reported increased fruit and vegetable consumption alongside reduced intake of ultra-processed foods. Although changes in dietary habits were observed, including an increase in fruit and vegetable consumption and a reduction in ultra-processed food intake, these were not the primary response variables of this study. The primary focus of the intervention was on physical activity levels, anthropometric changes, and overall body composition. Therefore, dietary data are reported descriptively to provide contextual support for the intervention’s outcomes but are not subjected to detailed statistical analysis in this study.

The change in dietary patterns, in combination with improved physical activity, contributed to a more comprehensive enhancement of health indicators, particularly among obese children with higher baseline sedentary levels and less healthy dietary habits. These outcomes highlight the intervention’s capacity to create meaningful behavioral changes, especially among children at greater risk, and underscore the program’s potential for scalable application to a national level. Future studies are recommended to explore the long-term sustainability of these outcomes and their impact on reducing childhood obesity in Chile.

## 4. Discussion

The findings from this study reaffirm the efficacy of community-based interventions in addressing childhood obesity, particularly through strategies targeting physical activity and nutritional education. The significant reduction in sedentary time among participating children is consistent with global evidence, underscoring the importance of introducing structured, age-appropriate physical activities during early childhood. These activities not only mitigate sedentary behaviors but also enhance motor skill development, psychosocial well-being, and overall physical fitness. Recent international research further supports the multifaceted benefits of early interventions, highlighting improvements in cognitive outcomes and social skills among children exposed to regular physical activity [44,45].

Programs in regions such as North America and Asia have demonstrated the adaptability and success of structured physical activity interventions, even in culturally and economically diverse settings. For instance, early childhood initiatives in South Korea and Canada have shown reductions in obesity rates and improvements in children’s physical health through integrated approaches that combine physical activity with family involvement [46,47]. Similarly, programs in Australia have emphasized the role of early education centers in fostering structured play environments, resulting in substantial health and developmental benefits [18,48].

Nutritional education formed a cornerstone of this study’s intervention, with notable dietary improvements observed in participating children and families. Increased fruit and vegetable intake and reduced consumption of ultra-processed foods align with global recommendations for promoting nutrient-dense diets to combat obesity [49]. However, the persistence of food deserts and limited access to healthy foods in underserved communities remains a significant barrier. Interventions incorporating parent-focused educational components have proven to be effective in addressing these disparities, as evidenced by programs in Brazil and the United States that have successfully enhanced dietary behaviors through community-based workshops and policy changes [15,50].

A critical achievement of this study was the significant reduction in adiposity, as evidenced by decreased skinfold thickness and body fat percentages among obese children. These findings align with meta-analyses demonstrating the effectiveness of combined dietary and physical activity interventions in reducing markers of adiposity and improving metabolic health outcomes in children [51,52]. Importantly, these reductions were more pronounced among children with higher baseline sedentary behaviors, emphasizing the need for targeted approaches tailored to at-risk populations.

Despite these successes, the modest changes in BMI and body weight highlight the multifactorial nature of childhood obesity and the challenges of achieving substantial weight reductions in the short term. Recent studies emphasize the necessity of long-term interventions that incorporate family engagement, follow-up support, and policy-level actions to address environmental and systemic contributors to obesity [53,54]. Moreover, individual variability in response to interventions—shaped by genetics, metabolic factors, and socio-environmental influences—necessitates personalized strategies to maximize effectiveness [55,56].

The broad applicability and scalability of this intervention are key strengths, demonstrating its potential for implementation across diverse socioeconomic and geographic settings. These findings support the global call for community-based, scalable interventions as a cornerstone of public health strategies to reduce childhood obesity [53]. The program’s success in Chile serves as a model for Latin America and other regions with similar demographic and health challenges, offering a blueprint for integrating cultural and contextual adaptations into health promotion efforts [57,58].

The broad applicability and scalability of this intervention are key strengths, demonstrating its potential for implementation across diverse socioeconomic and geographic settings within Chile and similar Latin American contexts. These findings support the need for localized, community-based interventions as a cornerstone of public health strategies to reduce childhood obesity. While the results may not be directly replicable in all international settings due to cultural and systemic differences, they offer valuable insights into the design of programs tailored to regions with comparable demographic and health challenges. The program’s success in Chile serves as a regionally relevant model, providing a framework for integrating cultural and contextual adaptations into health promotion efforts that could be applied to other Latin American countries facing similar epidemiological trends. Future research should explore the adaptability of this intervention to other contexts, considering policy variations, dietary patterns, and socioeconomic determinants that influence program effectiveness.

While this study demonstrates the effectiveness of a community-based intervention in improving physical activity levels, dietary habits, and body composition among preschool children, some limitations must be acknowledged. The quasi-experimental design, without randomization, may introduce selection bias, potentially limiting the generalizability of findings. Additionally, the significant difference in sample sizes between Phase 1 (n = 265) and Phase 2 (n = 939) resulted from the expansion of the intervention across multiple regions, which, while strengthening external validity, may have introduced regional variations that influenced outcomes. Another key limitation is the reliance on parental 24-h dietary recalls, which may be subject to recall bias despite cross-validation with daycare observations. The short-term follow-up also restricts conclusions about the long-term sustainability of behavioral and anthropometric improvements. Furthermore, although accelerometers provided objective physical activity data, compliance varied, and home activity patterns were not continuously tracked, potentially underestimating total daily movement. Lastly, external factors such as socioeconomic disparities and access to healthy foods may have influenced intervention effectiveness differently across study locations. Future research should incorporate randomized controlled trials, extended follow-ups, and strategies to enhance data accuracy, particularly in dietary reporting and physical activity monitoring, to further refine intervention strategies and ensure long-term impact.

While previous studies have identified parental education level and socioeconomic status as key determinants of childhood obesity [59], our study was conducted exclusively in public daycare centers serving low-income populations. This minimizes potential socioeconomic status disparities within the sample, reducing the risk of socioeconomic status-related confounding. Nonetheless, future studies should consider collecting direct measures of parental education and household income to further validate findings in diverse settings.

## 5. Future Research Directions

Future research should focus on evaluating the long-term sustainability of behavioral and health improvements observed in this study. While short-term behavioral changes in physical activity and diet were evident, longer follow-up studies are recommended to determine whether these improvements are maintained over time. Longitudinal studies are essential to understand the persistence of positive outcomes into adolescence and adulthood. Additionally, further exploration of the genetic, environmental, and psychosocial determinants of intervention efficacy can provide insights into personalized strategies for obesity prevention.

Expanding the use of technology, such as mobile health applications and wearable devices, could enhance participant engagement and data collection, offering real-time feedback and monitoring capabilities. Research on the integration of policy-level changes, such as increasing access to recreational facilities and subsidizing healthy food options, is also critical to complement community-based interventions. Finally, cross-cultural comparisons of similar programs could help identify universal best practices while allowing for regional adaptations.

By addressing these areas, future research can contribute to the development of comprehensive, sustainable solutions to reduce childhood obesity globally.

## 6. Conclusions

The reductions in BMI and body fat percentage observed in this study were modest; they align with findings from other early childhood interventions, where even small improvements in adiposity and physical activity have been associated with long-term health benefits [60]. Research suggests that early interventions targeting physical activity and dietary habits can contribute to improved metabolic health trajectories, even if short-term changes in anthropometric variables are limited [61]. Additionally, small changes at the population level can translate into significant public health benefits when scaled up, particularly in preventing further weight gain in at-risk populations [62]. Future studies with longer follow-up periods will be necessary to assess whether the behavioral changes induced by the intervention lead to more pronounced long-term health outcomes.

This study offers compelling evidence that community-based interventions designed to promote physical activity and healthy eating can meaningfully reduce childhood obesity among preschool-aged children. The significant improvements observed across three distinct Chilean cities underscore the efficacy and adaptability of the intervention methodology, demonstrating its potential for broader application at the national level. With institutional backing from the Ministry of Education and collaboration across local agencies, this validated approach holds promise for establishing sustainable and scalable health initiatives within public daycare settings.

The results are promising. Future research should prioritize longitudinal studies to assess the persistence of these behavioral and health improvements over time, particularly their influence on long-term obesity risk and overall health trajectories into adolescence and adulthood. Moreover, investigating the socioeconomic and environmental factors that may influence program effectiveness across diverse populations will be essential for optimizing and tailoring interventions. These findings highlight the potential of early, community-centered strategies to curb obesity rates and suggest that Chile’s approach could serve as a model for addressing childhood obesity in other Latin American countries and globally.

## Figures and Tables

**Table 1:** Initial anthropometric data of intervention participants and control children from three boroughs within Santiago, Chile.

	Intervention Participants (n = 120)	Control (n = 145)	*p*-Value
Age (y)	4.4 ± 0.4	4.5 ± 0.4	0.08
Weight (k)	18.6 ± 2.9	19.3 ± 3.1	0.06
Height (cm)	105.4 ± 4.6	106.6 ± 4.5	0.07
Weight-for-age Z-score	0.8 ± 1.2	0.7 ± 1.2	0.09
Height-for-age			0.05
Z-score	−0.1 ± 1.0	0.1 ± 1.0	
Weight-for-height			0.04 *
Z-score	0.8 ± 1.2	0.9 ± 1.2	

* Statistically significant differences at *p* < 0.05, *t*-test.

**Table 2:** Initial anthropometric data in Phase 1. Intervention participants versus control children from three boroughs within Santiago, Chile.

	Obese Children			Normoweight Children		
	Intervention Participants (n = 35)	Control (n = 42)	*p*-Value	Intervention Participants (n = 85)	Control (n = 103)	*p*-Value
Age (y)	4.4 ± 0.4	4.4 ± 0.4	0.85	4.4 ± 0.3	4.5 ± 0.4	0.06
Weight (k)	22.3 ± 2.5	23.6 ± 2.3	0.09	17.2 ± 1.7	17.8 ± 1.8	0.07
Height (cm)	107.2 ± 3.8	108.9 ± 3.8	0.07	104.7 ± 4.7	105.9 ± 4.5	0.09
Weight-for-age Z-score	2.2 ± 1.0	2.4 ± 0.9	0.11	−0.1 ± 0.7	−0.1 ± 1.1	0.87
Height-for-age						
Z-score	0.3 ± 0.8	0.3 ± 0.8	0.92	−0.3 ± 0.9	−0.2 ± 1.0	0.33
Weight-for-height						
Z-score	2.7 ± 0.8	3.0 ± 0.9	0.08	0.1 ± 0.5	0.3 ± 0.5 *	0.04

* Statistically significant differences at *p* < 0.05, *t*-test.

**Table 3:** Anthropometric data in Phase II. Intervention participants versus control children from three different cities in the North, South, and Metropolitan regions.

	Obese Children			Normoweight Children		
	Intervention Participants (n = 97)	Control (n = 86)	*p*-Value	Intervention Participants (n = 451)	Control (n = 305)	*p*-Value
Age (y)	4.5 ± 0.6	4.7 ± 0.6	0.07	4.5 ± 0.6	4.6 ± 0.5	0.08
Weight (k)	23.9 ± 3.1	23.4 ± 2.7	0.09	17.3 ± 1.8	17.3 ± 1.8	0.10
Height (cm)	107.1 ± 4.9	108.5 ± 5.0	0.05	104.5 ± 6.7	104.5 ± 4.9	0.07
Weight-for-age						
Z-score	2.5 ± 1.1	2.4 ± 0.9	0.06	−0.1 ± 0.7	−0.1 ± 0.8	0.09
Height-for-age						
Z-score	0.3 ± 1.0	0.4 ± 0.9	0.04 *	−0.2 ± 0.9	−0.4 ± 1.0	0.03 *
Weight-for-height						
Z-score	3.1 ± 1.1	2.9 ± 0.8	0.02 *	0.2 ± 0.5	0.2 ± 0.5	0.10

* Statistically significant differences at *p* < 0.05, *t*-test.

**Table 4:** Impact of the second phase on body fat (%) and the sum of skinfolds in both intervention participants and control children from the three cities.

Body Fat (%)	Intervention Participants			Controls		
	Pre	Post	*p*-Value	Pre	Post	*p*-Value
Total group	25.0 ± 4.6	24.7 ± 4.8	0.04 *	24.8 ± 4.4	25.1 ± 4.8	0.05 *
Obese	31.6 ± 4.3	31.6 ± 4.7	0.88	30.7 ± 4.0	31.4 ± 4.7	0.03 *
**Skinfolds**						
Sum of four skinfolds in obese children	60.3 ± 15.6	57.9 ± 20.4	0.02 *	52.6 ± 16.7	54.6± 19.6	0.05 *

* Statistically significant differences at *p* ≤ 0.05, paired *t*-test.

## Data Availability

Data is contained within the article.
